# RIG-I-like receptor activation drives type I IFN and antiviral signaling to limit Hantaan orthohantavirus replication

**DOI:** 10.1371/journal.ppat.1008483

**Published:** 2020-04-24

**Authors:** Alison M. Kell, Emily A. Hemann, J. Bryan Turnbull, Michael Gale

**Affiliations:** 1 Department of Molecular Genetics and Microbiology, University of New Mexico, Albuquerque, United States of America; 2 Department of Immunology, University of Washington, Seattle, United States of America; 3 Center for Innate Immunity and Immune Disease, University of Washington, Seattle United States of America; Thomas Jefferson University, UNITED STATES

## Abstract

Pathogenic hantaviruses, genus Orthohantaviridae, are maintained in rodent reservoirs with zoonotic transmission to humans occurring through inhalation of rodent excreta. Hantavirus disease in humans is characterized by localized vascular leakage and elevated levels of circulating proinflammatory cytokines. Despite the constant potential for deadly zoonotic transmission to humans, specific virus-host interactions of hantaviruses that lead to innate immune activation, and how these processes impart disease, remain unclear. In this study, we examined the mechanisms of viral recognition and innate immune activation of Hantaan orthohantavirus (HTNV) infection. We identified the RIG-I-like receptor (RLR) pathway as essential for innate immune activation, interferon (IFN) production, and interferon stimulated gene (ISG) expression in response to HTNV infection in human endothelial cells, and in murine cells representative of a non-reservoir host. Our results demonstrate that innate immune activation and signaling through the RLR pathway depends on viral replication wherein the host response can significantly restrict replication in target cells in a manner dependent on the type 1 interferon receptor (IFNAR). Importantly, following HTNV infection of a non-reservoir host murine model, IFNAR-deficient mice had higher viral loads, increased persistence, and greater viral dissemination to lung, spleen, and kidney compared to wild-type animals. Surprisingly, this response was MAVS independent *in vivo*. Innate immune profiling in these tissues demonstrates that HTNV infection triggers expression of IFN-regulated cytokines early during infection. We conclude that the RLR pathway is essential for recognition of HTNV infection to direct innate immune activation and control of viral replication *in vitro*, and that additional virus sensing and innate immune response pathways of IFN and cytokine regulation contribute to control of HTNV *in vivo*. These results reveal a critical role for innate immune regulation in driving divergent outcomes of HTNV infection, and serve to inform studies to identify therapeutic targets to alleviate human hantavirus disease.

## Author summary

Human pathogenic hantaviruses are endemic in rodent reservoir hosts worldwide. Incidental pathogenic human hantavirus infection has a case-fatality rate of up to 38%. Although Hantaan orthohantavirus (HTNV) is a major cause of human hemorrhagic fevers in Asia, the mechanisms underlying innate immune activation in human target endothelial cells remain unknown. Here, we find that the host cytoplasmic pathogen recognition receptors, RIG-I and MDA5, are essential for induction of antiviral proteins and control of HTNV replication in human and non-reservoir murine cells. Type I interferon, but not type III interferon, is induced in response to live virus infection, and can limit HTNV replication in these cells. We further demonstrate that type I interferon signaling is required for rapid control of virus replication and dissemination in non-reservoir murine hosts *in vivo*. Importantly, a role for type I interferon-independent mechanisms of antiviral induction *in vivo* was also revealed. This work provides deeper understanding of how differential host responses to HTNV infection contribute to infection outcomes and is essential to identify targets for therapeutic interventions to mitigate human hantavirus disease.

## Introduction

Hantaan orthohantavirus (HTNV) is the main causative agent of hemorrhagic fever with renal syndrome (HFRS) in humans, and is the most common etiology of hemorrhagic fevers in Asia. Human HTNV infection has a case-fatality rate as high as 10% [1, 2]. HFRS is characterized by elevated levels of proinflammatory cytokines and endothelial cell activation that leads to vascular leakage, thus linking HTNV infection and HFRS with underlying innate immune activation and inflammatory disease. Clinical research supports the hypothesis that HFRS is immune-mediated wherein innate immune activation and inflammation impart tissue damage and pathogenesis [3–6]. However, the mechanisms mediating virus recognition and innate immune activation in HTNV infection remain unclear.

HTNV is a member of the family Hantaviridae, tri-segmented, negative-sense, single-stranded RNA viruses in the order Bunyavirales. The three genome segments, referred to as small (S), medium (M), and large (L), encode four viral proteins; the viral nucleocapsid (N), the two viral surface glycoproteins (Gc and Gn), and the RNA-dependent RNA polymerase (L) [1, 7]. Around the world, hantaviruses have been identified in diverse reservoir hosts, but human pathogenic hantaviruses are, as yet, only found in rodent reservoir hosts [8, 9]. Zoonotic transmission of hantavirus to humans occurs via inhalation of aerosolized virus particles in rodent excreta. Although, the vascular endothelium is the primary cellular target of hantavirus infection in both humans and in reservoir hosts, infection drives very different outcomes of acute pathogenesis in humans while persistent, nonpathogenic infection occurs in reservoir hosts [10]. Hantavirus disease in humans begins with intense muscle pain, fever, and nausea, accompanied by elevated levels of the proinflammatory cytokines IL-1β, TNFα, and IL-6 and others [2, 11–13]. Vascular leakage, either in the lung microvasculature or kidneys, is the hallmark of disease and can be linked to lethal disease. Hantavirus infection of the endothelium is non-lytic, wherein the mechanisms underlying vascular leakage in human hantavirus infection are thought to include active antagonism of cell-cell adhesion molecules and induction of platelet activation factors that stimulate clotting and lymphocyte recruitment [10, 14, 15]. However, type I interferons (IFN) and proinflammatory cytokines, resulting from innate immune activation, can also increase vascular permeability and dysregulation of endothelial functions [16, 17], though the role of innate immune activation in catalyzing HFRS disease symptoms has not been understood. Further, the pathogen recognition receptor(s) (PRR) responsible for recognition and activation of the innate immune response during hantavirus infection of endothelial cells are not well-defined.

Endothelial cells, a major target cell of hantavirus infection, express cellular PRRs including the membrane bound Toll-like receptors (TLR) 3, 4, and 9, and cytosolic nucleic acid receptors cGAS [18] and the RIG-I-like receptors (RLR), RIG-I and MDA5. These PRRs bind to pathogen associated molecular patterns (PAMP) embedded within viral products. PAMPs include microbial lipids, proteins, and nucleic acid wherein PAMP recognition by PRRs drives signaling cascades resulting in innate immune activation [19]. TLRs initiate signaling through the adaptor proteins TRIF or MyD88, inducing NF-κB or interferon regulatory factor 3 (IRF3) transcriptional activity. Signaling through the RLR pathway occurs via interaction of the RLRs with MAVS on the mitochondrial associated membrane, leading to NF-κB and IRF3 activation [20]. NF-κB and IRF3 are essential for directing the production of type I and type III interferons (IFN) to mediate antiviral actions through the expression of virus-induced genes and interferon-stimulated genes (ISGs) whose products impart antiviral and/or immune modulatory function against infection. Early studies showed that overexpression of the ISG and antiviral effector MxA could inhibit replication of Old World hantaviruses Puumala and Tula virus, implicating IFN action through ISGs in defense against hantaviruses [21]. Moreover, HTNV infection induces IRF3 activation in human keratinocytes [22], wherein TLR and RLR-dependent signaling have all been reported to be required for innate immune activation against hantavirus in human cell lines [23–27]. Owing to a possible role for RIG-I in signaling innate immunity against hantaviruses, Mackow and colleagues demonstrated that the Andes virus glycoprotein could antagonize the RIG-I-dependent IRF3 activation in human cells to inhibit virus-activated gene expression and antiviral actions linked with IRF3 [28, 29]. However, it remains unclear which PRR(s) are required for hantavirus recognition in human endothelial cells.

Few tractable animal models exist to mimic hantavirus disease seen in humans. Historically, efforts to infect adult *Mus musculus* with Old World hantaviruses, Seoul virus (SEOV) or HTNV, led to asymptomatic infections and presumed viral clearance in female BALB/c [30, 31]. In contrast, newborn mice and SCID mice succumbed to virus infection but did not reproduce the vascular leakage or hemorrhagic disease typical of pathogenic human infection [31–33]. Recently, serial adaptation of a human HTNV isolate in newborn BALB/c mice has produced a model of hemorrhagic disease [34]. The Syrian hamster has been characterized most recently as the closest small animal model for human disease following infection with Andes virus and therefore has been the focus of intense study to identify the immune components contributing to pathogenesis and required for viral clearance [35–37]. Importantly, depletion of T cells or alveolar macrophages did not alter disease or Andes virus replication in this model [35, 38]. Similar disease in the hamster model following infection with other New World hantaviruses can be induced through immunosuppression of neutrophils, macrophages and other lymphocyte functions with the drug cyclophosphamide, but not with inhibitors of the NF-κB pathway and proinflammatory responses [39]. Together these studies suggest that innate immune activation and response link with pathogenic outcome in non-natural hosts of hantavirus infection. Conversely, recruitment of neutrophils and lymphocytes to the site of infection have been implicated in viral clearance in non-reservoir rodent hosts [40]. Thus, regulation of innate immune action is critical for the outcome of hantavirus infection.

In this study, we examined the role of TLR, RLR, and IFN signaling in directing innate immune activation against HTNV within *in vitro* and *in vivo* models of infection. Our studies identify major roles for RLR and type I IFN signaling in innate immune activation and virus control of HTNV infection in human endothelial cells and murine embryonic fibroblasts (MEFs) *in vitro*. We also reveal a role for type I IFN in directing innate immune activation and immune regulation for virus control *in vivo* within the non-reservoir murine model. These results demonstrate that HTNV infection initiates innate immune activation in human cells and non-reservoir murine hosts wherein type I IFN is required for immune programing and control of infection *in vivo*.

## Results

### HTNV replication drives innate immune signaling in human endothelial cells

**Fig 1:**
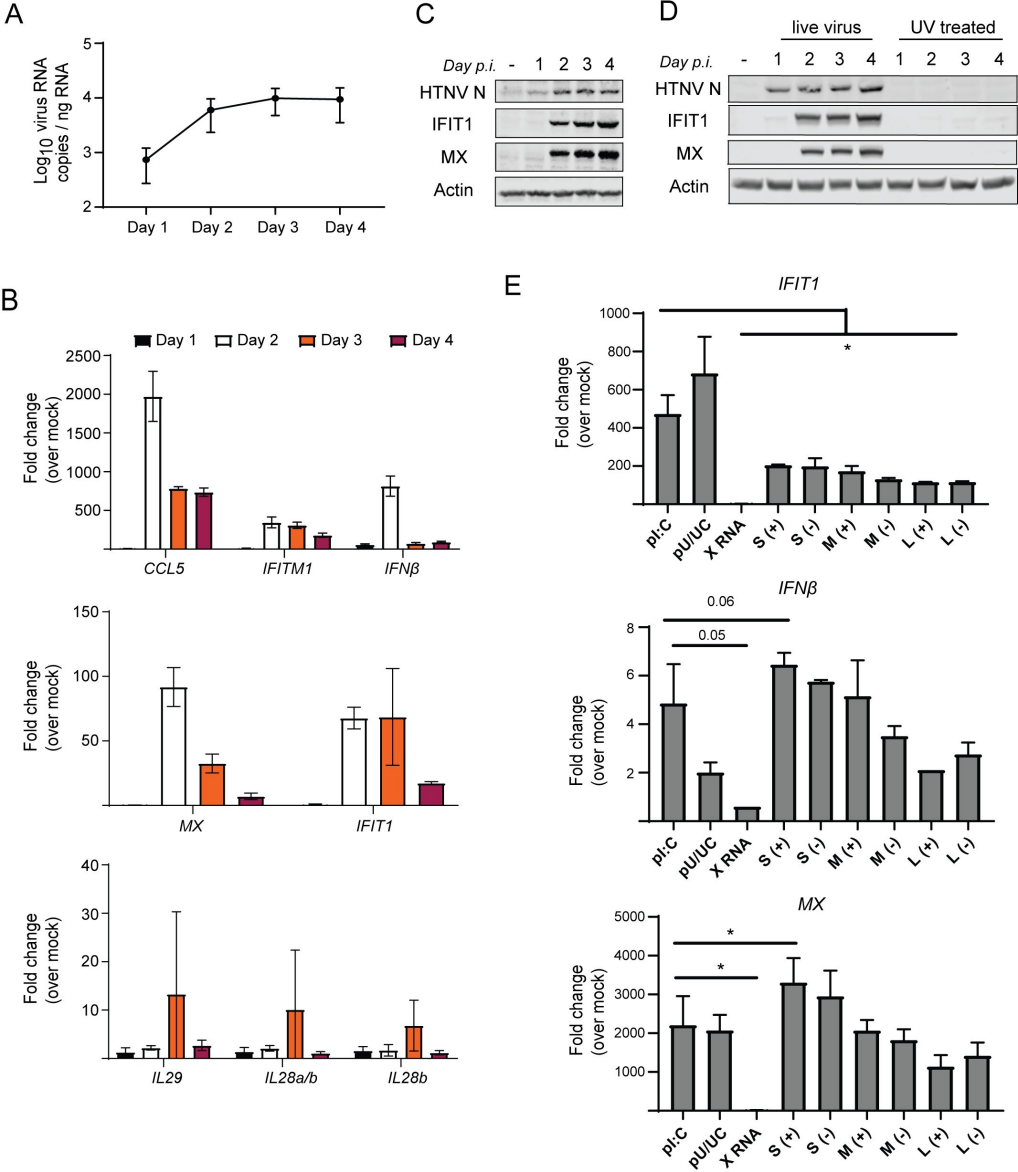
HTNV replication drives induction of antiviral and ISG responses in HUV-EC-C. HUV-EC-C were infected with HTNV (MOI 0.1) and harvested for RNA and protein analysis over 4 days post-infection (day p.i.). (A) qRT-PCR to quantify viral RNA copies (±SD). (B) Comparative gene expression analysis of indicated ISGs in HTNV-infected HUV-EC-C over mock infected HUV-EC-C (ΔΔC_t_) (±SD). (C) Western blotting analysis for host IFIT1, MX, and HTNV nucleocapsid (N) protein expression. (D) Western blotting analysis for HTNV N, IFIT1, and MX in HUV-EC-C exposed to either UV-inactivated or untreated HTNV (both at MOI 0.5), then harvested for 4 day p.i. (E) HepG2 cells were transfected with RLR stimulatory RNAs, HCV pU/UC and pI:C, or HTNV IVT RNA (S, M, L coding regions) to interrogate antiviral gene expression by RT-PCR (±SD). 1pmol of each RNA was transfected using Mirus TransIT transfection reagent and cells were harvested 18 hours later. (+) denotes positive sense transcript and (-) denotes negative sense transcript for HTNV segments. Data shown represent three independent experiments. Statistical analysis performed with one-way ANOVA to determine deviation from pI:C-induced expression levels, * denotes p adjusted < 0.05.

Endothelial cells are the primary target cells of HTNV infection in humans [41]. HTNV efficiently infects primary human endothelial cells *in vitro* (Fig 1A) and induces innate immune activation marked by induction of *IFIT1*, an IRF3-target gene and ISG [42], type I IFN (*IFNβ*) and ISG (*MX* and *IFITM1*) expression, as well as innate immune chemokine (*CCL5*) expression but does not induce type III IFN (*IL28A*, *IL28B* or *IL29*) expression (Fig 1B). Innate immune activation and the expression of antiviral proteins occurred concomitant with viral protein expression on day 2, indicating that HTNV replication links with innate immune activation in endothelial cells (Fig 1C). To determine if possible PAMPs within the incoming HTNV virion may induce innate immune activation, we treated cells with either live or UV-inactivated HTNV at a multiplicity of infection (MOI) of 0.5 and collected cells daily for 4 days post-treatment. In stark contrast to the live virus infected cells, we observed no induction of innate immune activation in cells exposed to UV-inactivated virus (Fig 1D), indicating that PAMP products of viral replication likely impart innate immune activation during HTNV infection. The RLRs recognize non-self RNAs in the cytoplasm following viral infection or cellular transfection with stimulatory RNA. The poly-U/UC motif from the 3’ untranslated region of the hepatitis C virus (pU/UC) is potently stimulatory for RIG-I activation, and polyinosine-polycytidylic acid (pI:C) activates both RIG-I and MDA5 when transfected into cells. To determine whether viral RNA could trigger this antiviral response, we *in vitro* transcribed (IVT) the positive (+) and negative (-) sense nucleotide sequences of the protein coding regions of each of the HTNV segments. We transfected equal molar amounts (1pmol) of HTNV S, M, and L along with known stimulatory RNAs HCV pU/UC and pI:C into HepG2 cells (Fig 1E and S1 Fig). X RNA is an equivalently sized RNA sequence found adjacent to the HCV pU/UC and previously shown not to stimulate RIG-I activation and signaling [43]. Because DNA/RNA transfection was found to be toxic for HUV-EC-C, HepG2 cells were used instead. We measured the induction of *IFNβ* and antiviral genes *MX* and *IFIT1* 18 hours after transfection with the IVT RNAs for each HTNV segment, and did not observe a significant difference in innate immune induction between the positive and negative sense RNAs. *IFIT1* induction was reduced in cells transfected with HTNV RNAs compared to pI:C and pU/UC RNA but comparable levels of *IFNβ* and *MX* were induced. In general, L segment transcripts induced lower levels of antiviral transcription. This may be in part due to the reduced integrity of the L segment transcripts as measured by denaturing agarose gel (S1 Fig). Importantly, IVT RNAs were not modified to trim the 5’-triphophate motif generated by T7 transcription and thus contain a required motif for RIG-I activation. However, hantavirus genomic RNAs have been reported to contain a 5’-monophosphate motif, possibly to evade RIG-I recognition [44]. Therefore, we conclude that sensing of HTNV gRNA or replication intermediates is likely driving innate immune activation in human endothelial cells, consistent with other reports [45].

### Type I IFN signaling restricts HTNV replication in endothelial cells

**Fig 2:**
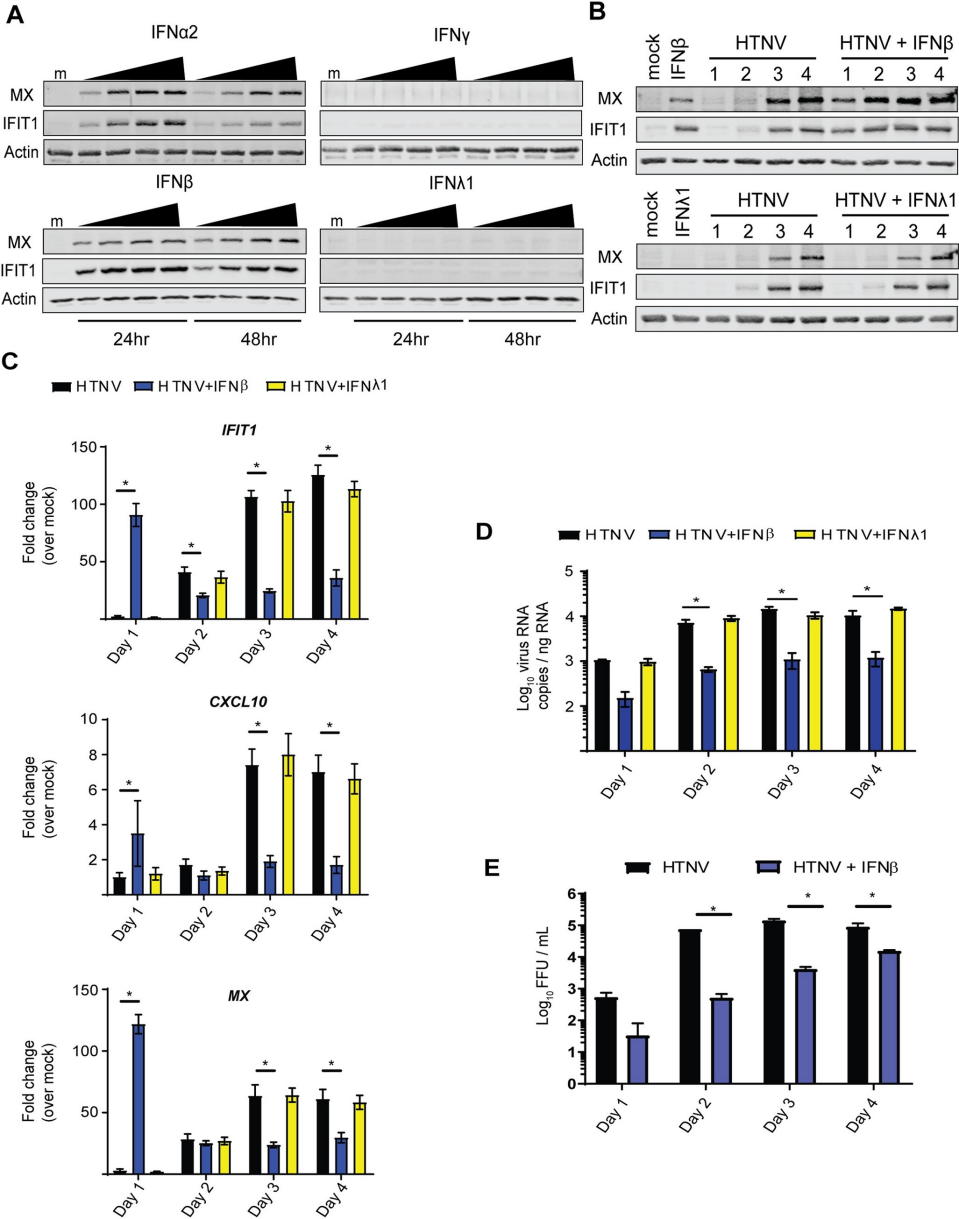
Type I IFN restricts HTNV replication in human endothelial cells. (A) HUV-EC-C were treated with culture media containing human recombinant IFNα2 (1000–15,000 U/mL), IFNβ (5–100 U/mL), IFNγ (0.1–7.5 ng/mL), or IFNλ1 (0.1–10 ug/mL). After 24 or 48 hours, ISG expression was assessed by western blotting. (B) HUV-EC-C were infected with HTNV (MOI 0.1) and then cultured in media containing 50 U/mL IFNβ or 10 ug/mL IFNλ1 for 4 days without changing media. Cells were harvested daily for western blotting (B) and RT-PCR (±SD) (C) analyses for gene expression, and qRT-PCR (±SD) (D) to quantify viral RNA. (E) Culture supernatants were assayed for HTNV FFU on vero cells. Statistical analysis performed with two-way ANOVA analysis, * denotes p adjusted < 0.05. Data shown represent three independent experiments.

To characterize the responsiveness of endothelial cells to IFN stimulation, we treated HUV-EC-C with increasing amounts of type I IFN (IFNα2, IFNβ), type II IFN (IFNγ), and type III IFN (IFNλ1) (Fig 2A). Type I IFNs, but not type II and III IFN, induced expression of ISGs IFIT1 and Mx beginning at 24hrs following treatment. Previous reports have suggested endothelial cells do not respond to type III IFN, likely due to very low type III IFN receptor (IFNλR) expression in this cell type [46]. To determine the effect of type I IFN on HTNV replication in endothelial cells, we infected HUV-EC-Cs with HTNV (MOI 0.1) and then, immediately following adsorption, cultured the infected cells in either standard media or media containing 50 U/mL human recombinant IFNβ or 10,000 ng/mL human recombinant IFNλ1. Analysis over 4 days post-infection demonstrated that treatment with type I IFN induced ISG expression earlier than in untreated infected cells (Fig 2B and 2C), and that this enhanced response was accompanied by a significant reduction in viral nucleocapsid (N) RNA and infectious virus produced (Fig 2D and 2E). Of note, IFNβ treatment appeared to limit virus replication and production following infection, but did not result in clearance of viral RNA through the infection time course and did not prevent continued virus release (Fig 2D and 2E). We therefore conclude that, in this system, type I IFNs are likely acting to prime uninfected cells and limit viral spread in culture. As expected, treatment with IFNλ after infection had no effect on ISG expression or HTNV replication. These results demonstrate the specific importance of type I IFN signaling and ISG actions to control HTNV infection in human endothelial cells, and verify the lack of type III IFN response in this specific cell type [46, 47].

### Innate immune signaling and viral control in HUV-EC-C requires RLRs

**Fig 3:**
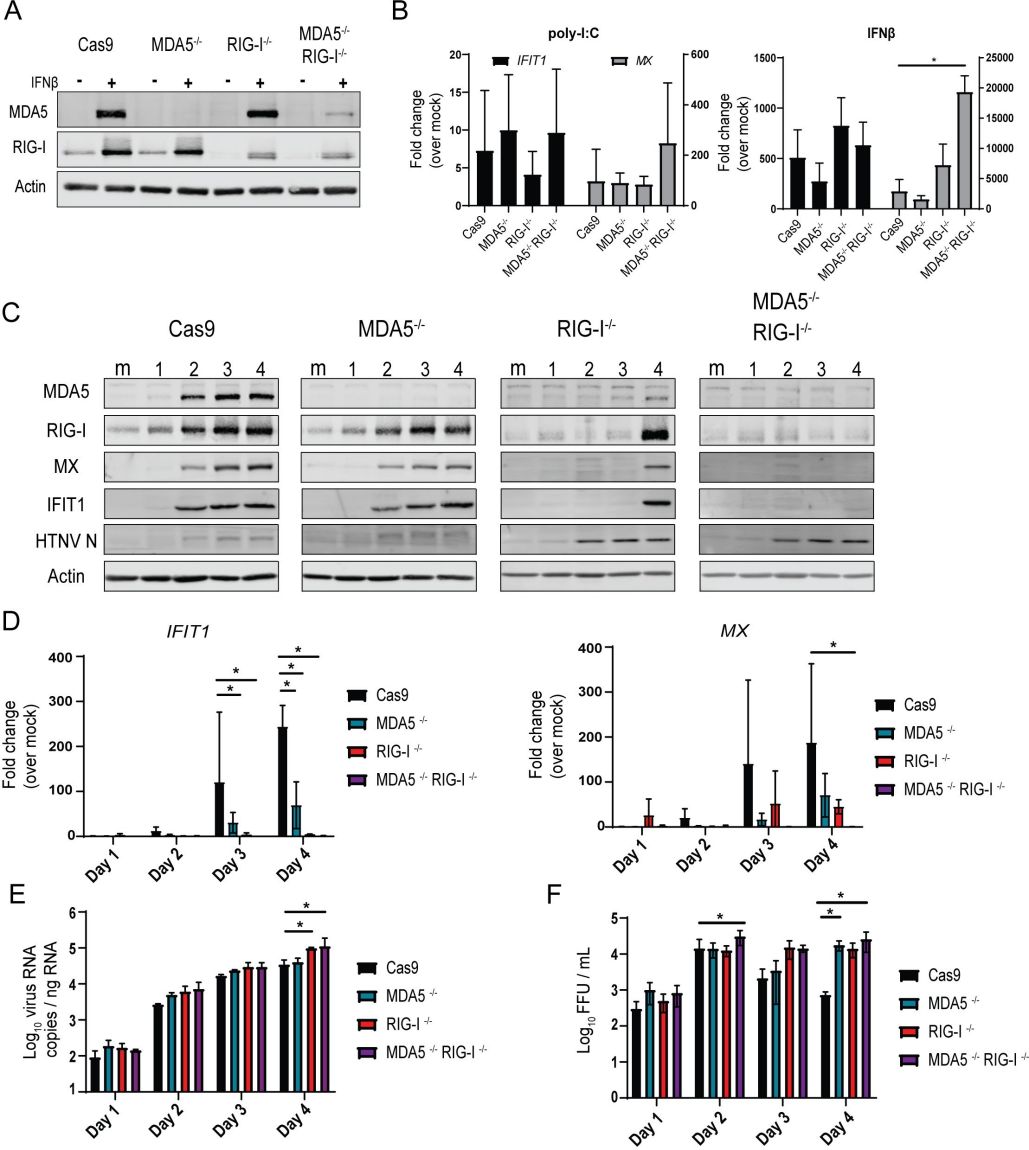
RLRs drive innate immune signaling and control HTNV replication. (A) Western blotting demonstrates specific knock-outs for CRISPR-Cas9 transgenic HUV-EC-C lacking MDA5 and RIG-I. Cells were either mock-treated or treated with 10U/mL IFNβ for 24hr to induce ISG expression. (B) RT-PCR analysis of ISG transcription in HUV-EC-C KO following treatment with exogenous pI:C (1pmol) or recombinant IFNβ (10U/mL) for 18 hours (±SD). (C) Western blotting analysis for HUV-EC-C KO cells following HTNV infection (MOI 0.1) harvested for four days p.i. (D) RT-PCR of ISG expression and (E) qRT-PCR pf HTNV nucleocapsid expression in HUV-EC-C CRISPR KO cells following HTNV infection (MOI 0.1) harvested for four days p.i. (±SD) (F) Viral titer quantification from supernatants of HTNV-infected HUV-EC-C KO cells (±SD). Data shown represent three independent experiments. Statistical analysis performed with two-way ANOVA analysis, * denotes p adjusted < 0.05.

To identify the catalyst of antiviral responses in infected endothelial cells, we assessed the role of the RLRs in triggering innate immune activation in HTNV infection. We created HUV-EC-C cells lacking RIG-I, MDA5, or both RIG-I and MDA5 using CRISPR-Cas9 technology [48] (Fig 3A). Immunoblot analysis shows specific knockout of the individual targeted proteins. RIG-I and MDA5 are themselves ISGs and their abundance increases in response to IFNβ treatment [49]. As reported for other cell lines, MDA5 is expressed at very low levels in unstimulated HUV-EC-C, but expression increases significantly following IFN signaling [50–53]. RLR expression was ablated in the respective knockout cell lines following 24 hr treatment with 10U/mL IFNβ (Fig 3A). Interestingly, following IFNβ treatment, RIG-I^-/-^ cells appear to express two lower molecular weight proteins recognized by our antibody (raised against amino acids 201–713). Our guide RNA for RIG-I was designed to target a sequence within exon 1 and thus may have resulted in the expression of a truncated RIG-I protein lacking one or both of the CARD signaling domains required for MAVS association [54]. To ensure that CRISPR knockout cells maintained intact type I IFN and TLR signaling, we treated cells with 10U IFNβ or 1 pmol pI:C to stimulate TLR3. All of the knockouts responded with increased ISG transcription, indicating that TLR and Jak/Stat signaling pathways remain intact in these cells (Fig 3B). Notably, pI:C and IFNβ treatment induced only low levels of *IL29* gene expression even in non-targeted control cells (Cas9) (S2 Fig). However, upon infection of the knockout lines with HTNV, we observed delay in HTNV-induced innate immune activation in the RIG-I^-/-^ compared to MDA5^-/-^ and Cas9, evidenced by delayed IFIT1 and MX protein and gene expression (Fig 3C and 3D). We observed a late accumulation of ISGs including the low molecular weight (potentially truncated) RIG-I in the RIG-I^-/-^ cells. ISG expression was completely ablated in the RIG-I^-/-^MDA5^-/-^ double knockout cells, suggesting that MDA5 may drive signaling late during infection even in the absence of RIG-I. Immunoblot analysis showed a greater accumulation of HTNV nucleoprotein in the RIG-I^-/-^ cells and RIG-I^-/-^MDA5^-/-^ cells compared to MDA5^-/-^ and control non-target cells (Fig 3C). Further, both RIG-I^-/-^ and RIG-I^-/-^MDA5^-/-^ HUV-EC-C contained significantly more cell-associated viral RNA (Fig 3E), and produced significantly more infectious virus than non-target control lines (Fig 3F). Again, MDA5 appears to play a role in the response and control of HTNV late in infection, as evidenced by increased virus production in MDA5^-/-^ cells only on day 4 post-infection. To account for potential changes in virus infectivity in CRISPR knockout cells, we titered our HTNV stock on each HUV-EC-C cell line and found no differences in FFU between the lines, although the titer on all HUV-EC-C lines was found to be about 1 log lower than Vero cells (S2 Fig). Together, these results suggest that RNA PAMP sensing and signaling through both RIG-I and MDA5, contributes to early viral recognition and innate immune activation that restricts HTNV replication in human endothelial cells.

### RIG-I-like receptor pathway is required for innate immune activation against HTNV in non-reservoir rodent cells

**Fig 4:**
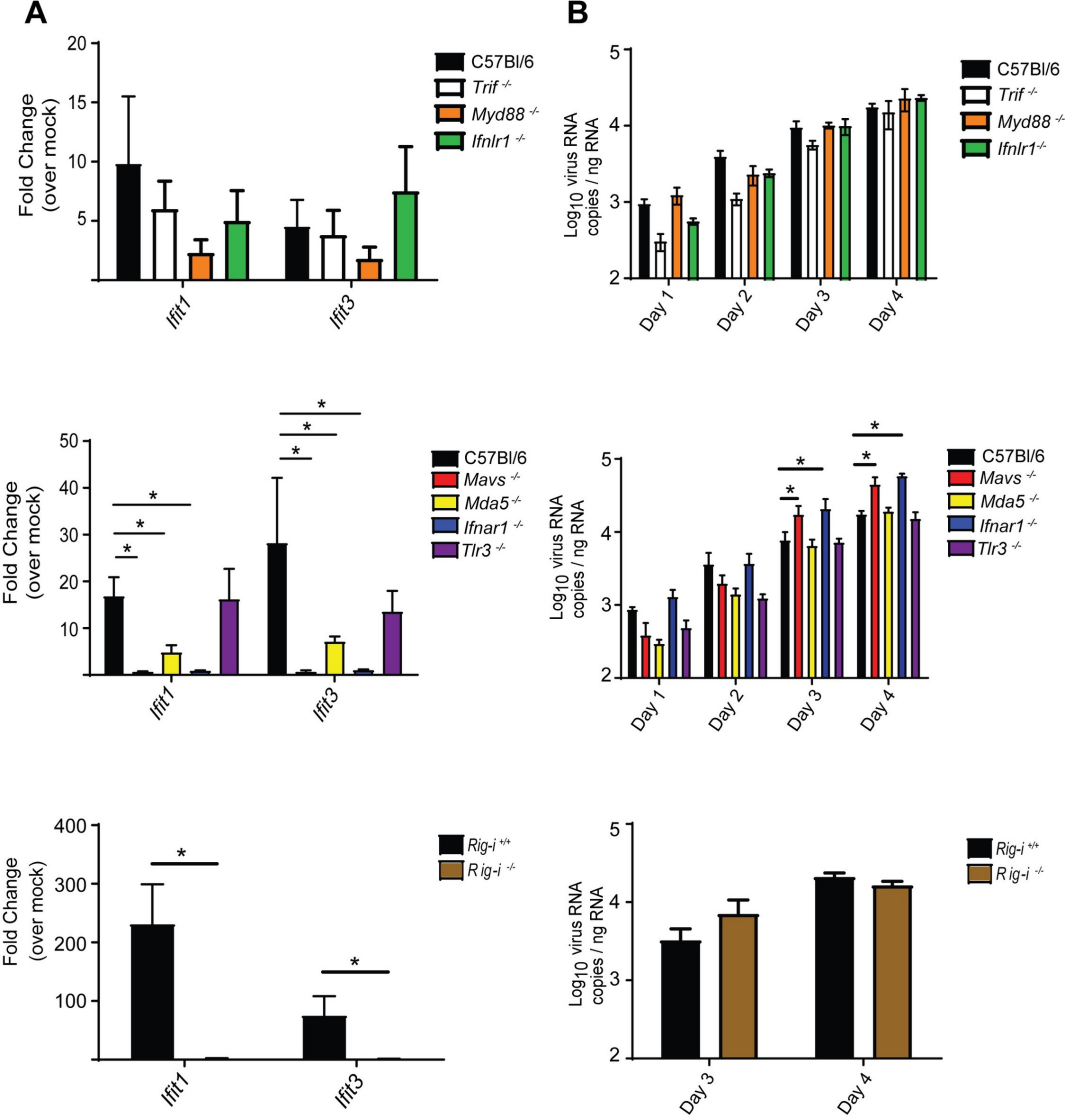
Transcriptional responses of MEFs to HTNV infection. Transgenic murine embryonic fibroblasts were infected with HTNV (MOI 1) and harvested for RNA for four days post-infection. Comparative (A) and quantitative (B) RT-PCR analysis was performed to determine antiviral gene expression and copies of viral nucleocapsid RNA, respectively (±SD). Comparative gene expression calculated as fold change over respective mock infected cells for each transgenic line on day 4 post-infection (ΔΔC_t_ ± SD). *Rig-i*^*+/+*^ and *Rig-i*^*-/-*^ cells were isolated from a B6/129/ICR mixed strain background. Three independent experiments were conducted in each of the groups shown. Statistical analysis performed with two-way ANOVA analysis, * denotes p adjusted < 0.05.

To further evaluate innate immune defenses against HTNV in diverse hosts, we examined the roles of cellular pathogen recognition receptors in initiating the innate immune response to HTNV infection in cells from a non-reservoir rodent host. For this purpose, we first assessed the response of primary murine embryonic fibroblasts (MEFs) from C57Bl/6J mice, including wild type (WT, C57Bl/6) mice and mice lacking MAVS, MDA5 (gene name *Ifih1*), the type I IFN receptor (*Ifnar1*), the type III IFN receptor (*Ifnlr1*) or the TLR signaling adaptor proteins TRIF and MyD88. (Fig 4). RIG-I (gene name *Ddx58*) knockout cells were isolated from a B6/129/ICR mixed strain background and were analyzed separately utilizing WT Rig-i+/+ controls on an appropriate matched background. Antiviral/ISG gene expression was only detectable by day 4 p.i., suggesting that these cells may be less susceptible to HTNV infection or that the virus employs a potent mechanism of immune antagonism (S3 Fig). HTNV infection in WT MEFs induced the expression of the antiviral genes Ifit1 and Ifit3 (Fig 4A, black bars). MEFs deficient for TLR signaling (*Tlr3*^*-/-*^, *Trif*^*-/-*^, *Myd88*^*-/-*^) and the type III IFN receptor (*Ifnlr*^*-/-*^) responded to HTNV infection by expressing ISGs to levels not significantly different from WT cells, although trends toward reduced signaling were observed for *Myd88*^*-/-*^ cells (Fig 4A). Further, these cells supported comparable levels of virus replication as quantified by cell-associated viral RNA (Fig 4B). In contrast, cells lacking either the type I IFN receptor (*Ifnar1*^*-/-*^) or MAVS failed to induce ISG expression upon infection (Fig 4A) and supported increased viral replication, indicating a dependence on RLR and type I IFN signaling for viral control (Fig 4B). While we consistently observed a significant reduction in ISG expression in cells lacking the individual RLR (*Mda5*^*-/-*^ and *Rig-i*^*-/-*^), virus replication was not significantly increased compared to WT, suggesting important, but potentially redundant, roles for each in controlling hantavirus replication in these cells. Consistent with our results in human endothelial cells, RIG-I may be involved in early innate immune signaling, with MDA5 playing a role later in infection, to drive antiviral responses and limit HTNV spread *in vitro*. Together, these results demonstrate a significant role for the MAVS-dependent RLR pathway to induce type I IFN signaling that is required to control viral replication in non-reservoir rodent cells.

### MAVS-independent type I IFN signaling is required for early control of virus replication *in vivo*

**Fig 5:**
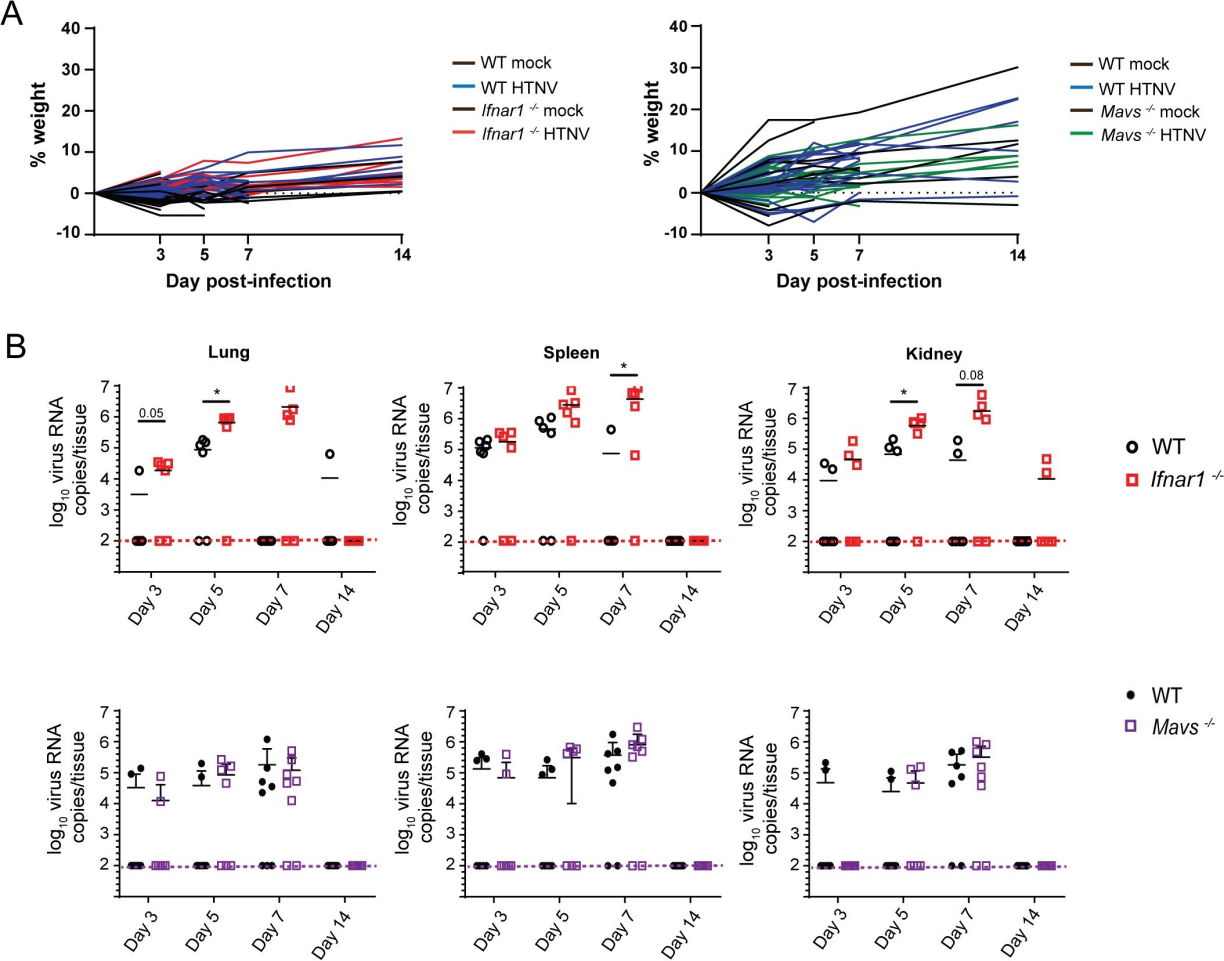
Type I IFN required for early control of HTNV replication and dissemination *in vivo*. C57BL/6J (WT), *Mavs*^*-/-*^ and *Ifnar1*^*-/-*^ mice were infected via intraperitoneal injection with 1x10^6^ FFU HTNV 76–118 or mock infected with PBS. Mice (n = 3/genotype) were euthanized on days 3, 5, 7, and 14 post-infection. Whole lung, spleen, and kidney tissues were collected in 0.5% BSA in PBS and homogenized. 25% of each homogenized tissue was subjected to TRIzol RNA extraction. (A) Animal weight and clinical scores for all WT (mock in black, virus infected in blue), *Ifnar1*^*-/-*^ (mock in black, virus infected in red), and *Mavs*^*-/-*^ (mock in black, virus infected in green). (B) Viral RNA was quantified by qRT-PCR and back-calculated as virus RNA copies/tissue (±SD). Each *in vivo* experiment was performed twice yielding n = 6 HTNV infected mice per genotype, per time point. Statistical significance determined by multiple T-tests using Holm-Sidak method, with alpha = 0.05, using Prism 8 software (* denotes p adjusted < 0.05).

Our *in vitro* results suggest that early RLR-dependent innate immune activation and type I IFN signaling are important for control of HTNV replication and production of infectious virus. To determine how RLR-dependent type I IFN signaling impacts innate immune control of HTNV infection *in vivo* in a non-reservoir host, we evaluated HTNV infection and innate immune response in WT, *Mavs*^*-/-*^ or *Ifnar1*^*-/-*^ C57Bl/6J mice as a non-reservoir host model of HTNV infection. Mice were mock-infected or challenged with 10^6^ FFU HTNV via intraperitoneal route, and tissues were collected 3, 5, 7, and 14 days post-challenge (Fig 5). Throughout the experiments, no signs of clinical disease or notable weight loss were observed in any of the animals (Fig 5A). Despite this, HTNV RNA was detected at higher quantities and at later time points in the *Ifnar1*^*-/-*^ mice compared to the WT control mice in all tissues (Fig 5B). In fact, while viral RNA did persist for many days in WT animals, we did not observe viral RNA increase over time in these animals, suggesting little or no significant infection. Instead, *Ifnar1*^*-/-*^ mice displayed statistically significant differences in viral load by day 5 in lung tissues, with all WT animals lacking detectable viral RNA by day 7 post-infection. The differences in viral replication between *Ifnar1*^*-/-*^ and WT animals was less pronounced in kidney and spleen tissues, although the trend was similar. In contrast, *Mavs*^*-/-*^ mice did not support increased HTNV replication or delayed viral clearance when compared to WT animals (Fig 5B). Thus, RLR-independent type I IFN defenses can restrict viral load and dissemination in lung, spleen and kidney early after HTNV infection in this non-reservoir rodent. Importantly, eventual viral clearance in *Ifnar1*^*-/-*^ mice suggests that type I IFN-independent immune defenses can also mediate HTNV clearance.

**Fig 6:**
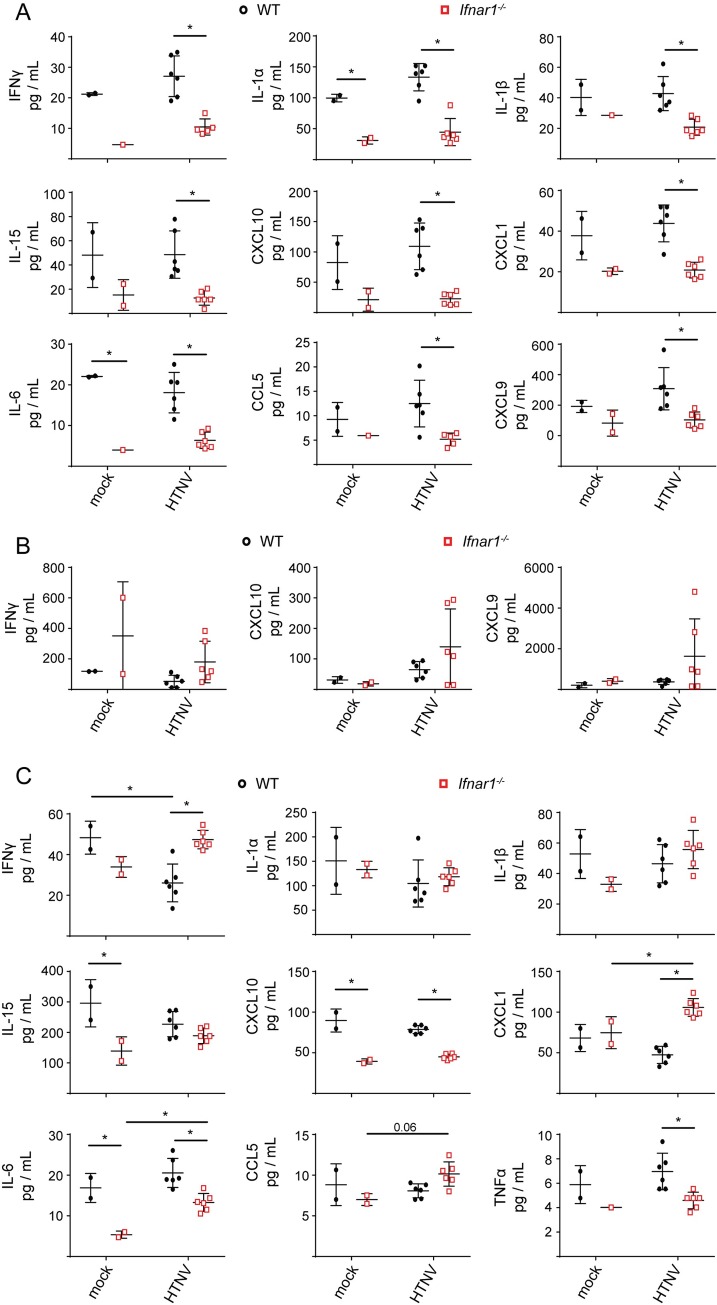
Cytokine/chemokine profiles of HTNV-infected WT and *Ifnar1*^*-/-*^ mice. Lung (A) and kidney (C) tissues from WT (black) and *Ifnar1*^*-/-*^ (red) mice, either mock-infected or infected with 10^6^ HTNV FFU, were assayed for protein expression on day 3 post-infection. (B) Protein expression in lung tissues from day 7 post-infection. Statistical analysis was performed with two-way ANOVA analysis with Prism 8 software, * denotes p adjusted < 0.05.

We also examined select cytokine and chemokine levels in lung and kidney tissues from WT and *Ifnar1*^*-/-*^ animals on day 3 post-infection in order to investigate the potential causes for the delay in viral clearance observed in *Ifnar1*^*-/-*^ mice. Expression of chemokines involved in immune cell recruitment to tissue sites of infection, CCL5, CXCL10, CXCL9, and IL-15, were significantly lower in lung tissues of HTNV-infected *Ifnar1*^*-/-*^ animals compared to WT controls (Fig 6A). IFNγ, and the proinflammatory cytokines IL-6 and IL-1α/β associated with human HFRS, were also reduced in *Ifnar1*^*-/-*^ mice. Importantly, on day 3, levels of these cytokines were lower in mock-infected *Ifnar1*^*-/-*^ as well, suggesting an intrinsic defect in these animals irrespective of infection status. By day 7 post-challenge however, levels of CXCL9, CXCL10, and IFNγ in *Ifnar1*^*-/-*^ animals trended towards an increase compared to WT animals (Fig 6B). The timing of this increase and the subsequent start of viral clearance leads us to hypothesize that immune cell trafficking could play an important role in HTNV clearance in the lungs of non-reservoir rodent hosts. In contrast, in kidney tissues, where we observed more subtle differences in viral load and clearance, CCL5, CXCL1, and IFNγ were increased in HTNV infected *Ifnar1*^*-/-*^ compared to WT controls (Fig 6C). Together, these results demonstrate that *Mus musculus* can support HTNV infection and viral replication, and suggests a link between a type I IFN-dependent early innate immune response and induction of immune chemokines and cytokines for rapid viral clearance.

## Discussion

Rapid recognition of virus infection and innate immune activation are critical to mount an effective antiviral response and to limit virus spread. Early innate immune signaling from the site of infection establishes the nature of the immune response and regulates the duration and intensity of immune activation [49]. In this study, we reveal an essential role for type I IFN signaling for early control of HTNV replication *in vitro* and *in vivo*. Moreover, we show that RLR signaling is important in driving this response *in vitro*, and that additional mechanisms also contribute to HTNV-induced innate immune activation and viral clearance *in vivo*.

Our results demonstrate that the HTNV RNA is likely driving ISG expression *in vitro* during infection. UV-inactivated virus failed to induce ISG expression *in vitro*, indicating that exposure to virion proteins, lipids or nucleocapsid-coated viral genomic RNA is not sufficient for innate immune activation. Instead, IVT RNAs for individual HTNV segments (S, M, and L) stimulated an antiviral transcriptional response when transfected into cells. Therefore, we hypothesize that uncoated RNA during viral gene transcription or replication intermediates likely serve as PAMPs of RLR recognition to trigger innate immune activation during HTNV infection. Further work to define the specific RNA sequences or motifs that serve as PAMPs for RIG-I and MDA5 in HTNV infection remains a priority for understanding the mechanisms of innate immune activation and control of HTNV infection in human endothelial cells.

We observed increased HTNV replication in the RIG-I^-/-^ and MDA5^-/-^RIG-I^-/-^ double knockout HUV-EC-C, thus supporting a role for the RLR pathway in innate immune control of infection. Interestingly, while the MDA5^-/-^ cells responded to HTNV infection with induction of Mx and IFIT1 protein expression similar to non-targeted control cells, a dramatic delay in ISG expression was observed in the RIG-I^-/-^ cells and completely absent in the double knockouts. This outcome suggests that the RLRs have non-redundant, but temporally distinct, roles in HTNV sensing, with RIG-I driving early immune signaling and MDA5 becoming involved later during infection. Similar conclusions have been drawn for RLR actions in recognizing and controlling flavivirus infection [50]. Our observations from the *in vitro* non-reservoir murine model also support the conclusion that RLR signaling through MAVS and type I IFN are fundamental to HTNV control. We observed a complete ablation of antiviral responses in MEFs lacking either *Mavs* or *Ifnar1*, showing that MEFs recapitulate innate immune response dynamics of early-stage HTNV infection. Surprisingly, in contrast to previous reports, we did not observe statistically significant reductions in gene expression or viral RNA in MEFs lacking TLR3 or the TLR adapter proteins MyD88 or TRIF [26, 27, 55]. However, we did observe a consistent trend towards a reduction in ISG expression in *Myd88*^*-/-*^ MEFs and increased viral RNA. Cell type differences (fibroblasts vs. endothelial cells) and host-specific virus interactions may explain these conflicting observations. Future studies will be required to define the mechanisms of respective PRR activation in different cell types and to determine how differential antiviral induction may determine disease outcome or virus control in each host.

IFNβ treatment of HTNV-infected human endothelial cells induces immediate ISG expression and hinders virus production, showing that type I IFN signaling can drive an effective response to limit HTNV spread in human target cells. Interestingly, although type III IFN has been shown to be induced upon HTNV infection of human epithelial cells and African green monkey cells, we found that neither *IL-29*, *IL-28A*, or *IL-28B* were transcriptionally induced in HTNV-infected or exogenously stimulated HUV-EC-C [56]. Moreover, consistent with previous reports, we show here that human endothelial cells also do not respond to IFNλ treatment to induce ISG expression or to restrict HTNV replication [46]. We conclude that human endothelial cells therefore rely upon type I IFN signaling for ISG expression and robust antiviral responses to HTNV.

Due to the importance of RLR signaling and type I IFN in HTNV control *in vitro*, we hypothesized that mice deficient for MAVS and the IFNα receptor may be more susceptible to HTNV infection and would lack systemic viral control. Indeed, *Ifnar1*^*-/-*^ mice had higher viral load in all tissues examined, with viral RNA in *Ifnar1*^*-/-*^ mice present late through infection in contrast to WT controls. Interestingly, in addition to intrinsic differences in cytokine expression in uninfected *Ifnar*^*-/-*^ animals, reduced expression of immune cell chemoattractants was observed on day 3 post-infection for *Ifnar1*^*-/-*^ mice compared to WT controls during HTNV infection. But, by day 7 this trend was reversed for IFNγ, CXCL10, and CXCL9. The role of immune cell infiltration to sites of hantavirus infection in mediating viral clearance remains an outstanding question and will require further study. Interestingly, this chemokine profile is not associated with any external signs of disease during HTNV infection. Increased levels of circulating proinflammatory cytokines, such as IL-6 and TNFα, are associated with more severe HFRS in human patients [57–59]. However, while we observed differences in basal expression levels between WT and *Ifnar1*^*-/-*^ animals, we did not observe statistically significant changes in these cytokines in HTNV-infected mice compared to mock-infected controls. A lack of strong signaling in HTNV-infected WT animals may be explained by the absence of increased viral RNA over time in these animals, suggesting low levels of active viral replication. Studies of reservoir host responses to the related *Seoul orthohantavirus* (SEOV) yielded similar observations of a lack of induction of proinflammatory cytokines even in the presence of high viral loads [60]. This outcome may therefore also represent a universal rodent host response to orthohantaviruses. The absence of proinflammatory responses, correlating with asymptomatic infections in our non-reservoir host mouse model, strengthens the evidence that hantavirus disease is driven by inflammation [9, 61]. Importantly, we observed no differences in viral load or tissue dissemination between WT and *Mavs*^*-/-*^ mice, indicating that other immune signaling pathways are involved in type I IFN production and viral control *in vivo*. Similarly, while we did detect increased and sustained HTNV RNA in the *Ifnar1*^*-/-*^ mice, all but two animals were able to clear the infection by 14 days, demonstrating that type I IFN is not the only mediator of viral defense in non-reservoir rodents. This intriguing result now opens the possibility that type I IFN-independent mechanisms of immune-mediated viral clearance control HTNV infection in *Mus musculus* hosts.

Our observations that WT mice clear the HTNV infection in the absence of disease is supported in the literature [31]. However, Wichmann *et al*. reported that HTNV infection caused lethal neurologic disease in four separate strains of WT mice, even at doses as low as 10^3^ pfu [62]. Further, in their hands, *Ifnar1*^*-/-*^ animals, on a C57BL/6 background, succumbed to disease significantly faster than C57BL/6 WT animals. It may be notable that female mice were used exclusively for the Wichmann study while our study was heavily weighted with male mice for HTNV infection. These inconsistencies in the differential outcome of HTNV infection could be due to potential sources of variance in mouse colonies, including genetic differences between WT strains and/or gender differences in the immune response to HTNV infection, as well as specific virological differences in HTNV strains, each requiring further study to resolve.

In summary, we demonstrate that RLR signaling through MAVS is required for control of HTNV replication through induction of ISGs and antiviral gene expression in human endothelial cells and mouse cell culture models. *In vivo*, in non-reservoir murine hosts, HTNV infection drives early type I IFN-dependent chemokine and cytokine expression, in which type I IFN-independent processes contribute to viral clearance. Our study reveals that ISGs and virus-induced genes serve as antiviral effectors for the control of HTNV infection and disease.

## Materials and methods

### Viruses and *in vitro* infections

Hantaan virus strain 76–118 was kindly provided by Drs. Sabra Klein and Andrew Pekosz and used for all virus infections described in this study. HTNV was propagated on Vero E6 cells (ATCC, CRL-1586) for 7 days. Infectious virus was isolated by harvesting cellular supernatant and spinning at 1000 rpm for 10 minutes to remove cellular debris. For virus infections, cells were cultured 18–20 hours at desired density in appropriate culture media. HTNV was added at desired MOI diluted in culture media to cellular monolayers for 2 hours at 37°C. Cells were subsequently washed once with PBS and appropriate culture media was added for the duration of the experiment. Mock infected cells were treated with culture media in the absence of virus. To inactivate virus stocks or tissues from infected animals, ultraviolet radiation was applied at 3 J/cm^2^ using a Stratalinker 2400 model crosslinking box. UV inactivation was validated by FFU assay on Vero E6 cells.

### Cell culture

Vero E6 cells (ATCC, CRL-1586) were cultured in Dulbecco’s modified Eagle's medium (DMEM) supplemented with 10% heat-inactivated FBS, 1% pen/strep, 2mM L-glut, 1% non-essential amino acids, 1% HEPES. Murine embryonic fibroblasts were isolated as previously described [50] and cultured in supplemented DMEM as described for Vero. Human umbilical vascular endothelial cells (HUV-EC-C) (ATCC, CRL-1730) were cultured in EGM-Plus Bullet Kit (CC-4542 + CC-5036) (Lonza, CC-5035) prepared according to manufacturer specifications. HUV-EC-C were cultured on tissue culture-treated plastics, coated with rat tail collagen (Corning, CB-40236).

For CRISPR-targeting of the indicated genes, DNA oligos containing off target or targeting gRNAs were inserted into a Cas9-t2a-puro pRRL vector using the In-Fusion HD Cloning Kit (Clontech) [63]. See Table 1 for guide RNA template sequences. Lentivirus pseudotyped with vesicular stomatitis virus envelope glycoprotein (VSV-G) was produced by transfection of 2x10^6^ HEK293T cells with the ProFection Mammalian Transfection System (Promega), 6ug gRNA-Cas9-t2a-puro pRRL lentivirus plasmid, 3ug psPAX-2 packaging plasmid and 1.5ug pVSV-G in 10cm plates for 48 hours before filtration of infectious supernatants with a 0.45uM filter. Target cells were transduced with the filtered viral supernatants for 24 hours, washed and cultured in fresh media for 24 hours before selection with 400ng/ml Puromycin or 2ug/mL Blasticidin. Cells were passaged in bulk in selection media. Targeting was evaluated by immunoblot and ablated signaling downstream of relevant stimuli.

**Table 1:** CRISPR guide RNA template sequences.

Target	Domain	gDNA sequence
Non-target		GACGGAGGCTAAGCGTCGCAA
MDA5	Exon 1	CGCACATTTCACCTGTCCCGCA
RIG-I	Exon 1	CCGCCGCTAGTTGCACTTTCGA
MAVS	Exon 4	CTCATTGCAGAATTCAGAGCAAGCC

Recombinant human IFNλ/IL29, IFNα2, and IFNγ were purchased from R&D Systems (1598IL025CF, 285-IF-100, 11105–1). Recombinant human IFNβ was provided by Toray Industries.

### Focus-forming unit assay

Infectious virus was quantified using immunostaining as previously described [64]. Briefly, Vero cells were infected by incubation with 100uL culture supernatants for 1 hour at 37°C. After incubation, a 0.5% agarose overlay was added, containing a 1:1 mixture of supplemented DMEM and 1% agarose in water. Cells were then incubated for 7 days at 37°C. After fixation of cells with 95% EtOH:5% acetic acid, cells were probed with primary (α HTNV nucleocapsid 76–118, BEI resources NR-12152) and secondary (donkey α rabbit IgG, HRP-conjugated, Jackson Immunoresearch 711-035-152) antibodies. SeraCare TrueBlue peroxidase substrate (VWR, 95059–168) was used to visualize and count antibody-positive foci in the cell monolayer on light microscope.

### RNA methods

RNA was isolated from cells treated with TRIzol Reagent or TRIzol LS Reagent (ThermoFisher Scientific, 15596026, 10296010). RNA to be used for gene expression analysis and virus copy quantification was extracted from TRIzol reagent using the Zymo Research Direct-zol RNA Miniprep Plus kit and quantified on a BioTek Epoch Microplate Spectrophotometer. cDNA was synthesized using the iScript^™^ Select cDNA Synthesis Kit (BioRad, 1708897BUN) by adding 400ng total RNA to a reaction mixture containing random hexamer primers as specified by the manufacturer. Real-time PCR was performed using the SYBR Green PCR Master Mix (ThermoFisher Scientific, 4312704) and the primers described Table 2. Gene specific primers were purchased from Integrated DNA Technologies or Qiagen. Host antiviral and ISG gene expression was normalized to the expression of mouse *Chmp2a* or human *RPL13A* mRNA. HTNV nucleocapsid copies were calculated by using a DNA plasmid standard and the noted primers during RT-PCR. Results were analyzed and graphed using Prism 8 software (GraphPad).

**Table 2:** Primer sequences for RT-PCR.

Species	Target	Sequence
human	Rpl13a	GCCCTACGACAAGAAAAAGCG
		TACTTCCAGCCAACCTCGTGA
human	Cxcl10	GTGGCATTCAAGGAGTACCTC
		TGATGGCCTTCGATTCTGGATT
human	Ifit1	AGAAGCAGGCAATCACAGAAAA
		CTGAAACCGACCATAGTGGAAAT
human	Ifitm1	TACTCCGTGAAGTCTAGGGACAG
		AACAGGATGAATCCAATGGTCA
human	Ifnβ	GGAGATGACGGAGAAGATGC
		CCCAGTGCTGGAGAAATTGT
human	Ccl5	Qiagen RT^2 qPCR Primer Assay
		
human	Mx	GTTTCCGAAGTGGACATCGCA
		CTGCACAGGTTGTTCTCAGC
human	IL29	Qiagen SABiosciences (PPH05849A)
		
human	IL28AB	Qiagen SABiosciences (PPH05847B-200)
		
human	IL28B	AAGGACTGCAAGTGCCGCT
		GCTGGTCCAAGACATCCC
mouse	Chmp2a	AGACGCCAGAGGAACTACTTC
		ACCAGGTCTTTTGCCATGATTC
mouse	Ifit1	CTGAGATGTCACTTCACATGGAA
		GTGCATCCCCAATGGGTTCT
mouse	Ifit3	TCAGGCTTACGTTGACAAGGT
		CACACTTTAGGCGTGTCCATC
HTNV	nucleocapsid	AAGCATGAAGGCAGAAGAGAT
		TAGTCCCTGTTTGTTGCAGG

The synthetic dsRNA poly(I:C) was purchased from InvivoGen. The hepatitis C virus (HCV) xRNA and polyU/UC RNA were *in vitro* transcribed from synthetic DNA oligonucleotide templates (Integrated DNA Technologies) using the T7 MEGAshortscript kit (Ambion) as previously described [43, 53, 65]. Primers used for PCR and oligonucleotides for pU/UC and X RNA synthesis can be found in Table 3. HTNV segment RNAs were in vitro transcribed from PCR templates using the T7 MEGAscript kit (Ambion) according to manufacturer’s instructions. RNA integrity was assessed with 2% formaldehyde-agarose gel and ethidium bromide staining (S1 Fig). RNA ladder or IVT RNA was mixed with RNA loading dye containing ethidium bromide (Life Technologies, High Range RuboRuler), heated to 80°C for 10 minutes, placed immediately on ice, and then run at 30V in a formaldehyde-agarose gel. Bands were visualized on BioRad ChemiDoc imaging system.

**Table 3:** Primers for IVT RNA synthesis.

Target	Sequence
HTNV N positive sense	TAATACGACTCACTATAGGGATGGCAACTATGGAG
	ATGGCAACTATGGAGGAATTAC
HTNV N negative sense	TCATTAATTAGAGTTTCAAAGG
	TAATACGACTCACTATAGGGTTAGAGTTTCAAAGGC
HTNV M positive sense	TAATACGACTCACTATAGGGATGGGGATATGGAAGTGGCT
	GTGTCTAGAGCGGCCCTATGATTTTTTATGCTTCCTTACG
HTNV M negative sense	CGATGACAAGGAATTCACCATGGGGATATGGAAGTGGCTAGTG
	TAATACGACTCACTATAGGGCTATGATTTTTTATGCTTCC
HTNV L positive sense	TAATACGACTCACTATAGGGATGGATAAATATAGAG
	GTCACGTGTCTAGAGCGGCCCTAATAGAAAGAGGAAATAGAATCC
HTNV L negative sense	GATGACGATGACAAGGAATTCACCATGGATAAATATAGAGAAATTCACA
	TAATACGACTCACTATAGGGCTAATAGAAAGAGG

### Protein analyses

Cell lysates to be analyzed for protein expression by western blotting were harvested in protein lysis buffer and prepared as previously described [51]. Antibodies used were specific for human proteins RIG-I (Adipogen, AG-20B-0009-C100), ISG56 (from G. Sen at Cleveland Clinic Foundation, Cleveland), Mx-1 (W. Lai, Antibody Production Core, UT Southwestern Medical Center), MAVS (Enzo Life Sciences, ALX-210-929-C100), MDA5 (IBL America, 29020), HTNV nucleocapsid 76–118 (BEI resources, NR-12152), and actin (Santa Cruz, SC-1616). All secondary antibodies were obtained through Jackson ImmunoResearch and visualized on a Licor Odyssey CLx imager. Images were cropped and uniformly modified using the Image Studio imaging software and Adobe Illustrator. Densitometry was performed using ImageJ software (NIH) and calculated as the relative density of HTNV nucleocapsid protein over actin. Cytokine/chemokine analysis was performed on UV-treated, homogenized mouse tissues using the Milliplex MAP mouse cytokine/chemokine magnetic bead panel kit (Millipore, MCYTOMAG-70k) run on a Bio-Rad Bioplex 200 Multiplexing Analyzer System according to manufacturer instructions. Results were analyzed and graphed using Prism 5 software (GraphPad).

### *In vivo* methods

Transgenic mice were raised in the Gale laboratory colony at the University of Washington and C57Bl/6J mice were ordered to be age and sex-matched from the Jackson Laboratory. For each experiment, 5–8 week old C57BL/6J mice (n = 20), *Mavs*^*-/-*^ (n = 20), or *Ifnar1*^*-/-*^ (n = 20) transgenic knockout mice were either infected (n = 16/strain) with 10^6^ FFU HTNV diluted to 100uL in PBS or mock infected (n = 4/strain) with an equal volume (100uL) of PBS via intraperitoneal injection. Two replicate experiments were performed for each knockout independently. In total, 122 male and 38 female mice were used. On days 3, 5, 7, and 14 post-infection, 4 mice/strain (3 HTNV-infected, 1 mock) were euthanized and lung, kidney, and spleen tissues were collected in PBS plus 0.5% BSA in Precellys tubes. The lungs of all mice were subjected to PBS perfusion prior to collection. Tissues were then processed using a Precellys homogenizer, centrifuged, and supernatants were aliquoted and stored at -80°C until downstream analysis. C57BL/6J animals were acquired from the Jackson Laboratory and transferred immediately, along with in house-reared transgenic animals, to an Animal Biosafety Level 3 enhanced containment facility 5 days before challenge. Animals were monitored for weight loss and clinical signs according to IACUC regulations and approved protocols at the University of Washington. Transgenic mouse lines in our colony are regularly genotyped to verify KO.

### Ethics statement

All animal experiments were conducted according to the animal use and care as specified on protocol #4158–03 (BSL3 studies) and #4158–01 (colony maintenance), which have been reviewed and approved by the University of Washington Institutional Animal Care and Use Committee (IACUC). This protocol adheres to the recommendations in the Guide for the Care and Use of Laboratory Animals as adopted by the Office of Laboratory Animal Welfare in the National Institutes of Health.

## Supporting information

S1 Fig Integrity of in vitro transcribed RNAs. (A) IVT RNA from HTNV segment DNA templates run on a denaturing agarose gel. (+) denotes positive sense transcript and (-) denotes negative sense transcript. (B) IVT pU/UC and X RNA sequences from hepatitis C virus prepared from DNA oligonucleotides and run on a denaturing agarose gel.
(TIF)

S2 Fig Type III IFN gene expression and susceptibility to HTNV infection in HUV-EC-C CRISPR knockout cell lines. (A) RT-PCR for *Il29* in HUVEC CRISPR lines treated with 1 pmol pI:C or 10U/mL IFNβ for 18hrs (±SD). (B) Titer for HTNV virus stock on HUV-EC-C CRISPR lines and Vero E6 cells. Data represent three independent experiments. Statistical analysis performed by two-way ANOVA with Prism 8 software.
(TIF)

S3 Fig Antiviral signaling in HTNV-infected MEF is not detected until day 4 post-infection. RT-PCR for Ifit3 and Ifit1 antiviral genes in WT and transgenic MEFs analyzed daily for four days post-infection with HTNV MOI 1 (±SD). (A) and (B) represent pooled data from different sets of triplicate experiments.
(TIF)
