# Supplementary figures and images for: RIG-I-like receptor activation drives type I IFN and antiviral signaling to limit Hantaan orthohantavirus replication
Source: PLoS Pathog. 2020 Apr 24;16(4):e1008483. doi: 10.1371/journal.ppat.1008483 (PMC7202661; doi:10.1371/journal.ppat.1008483)

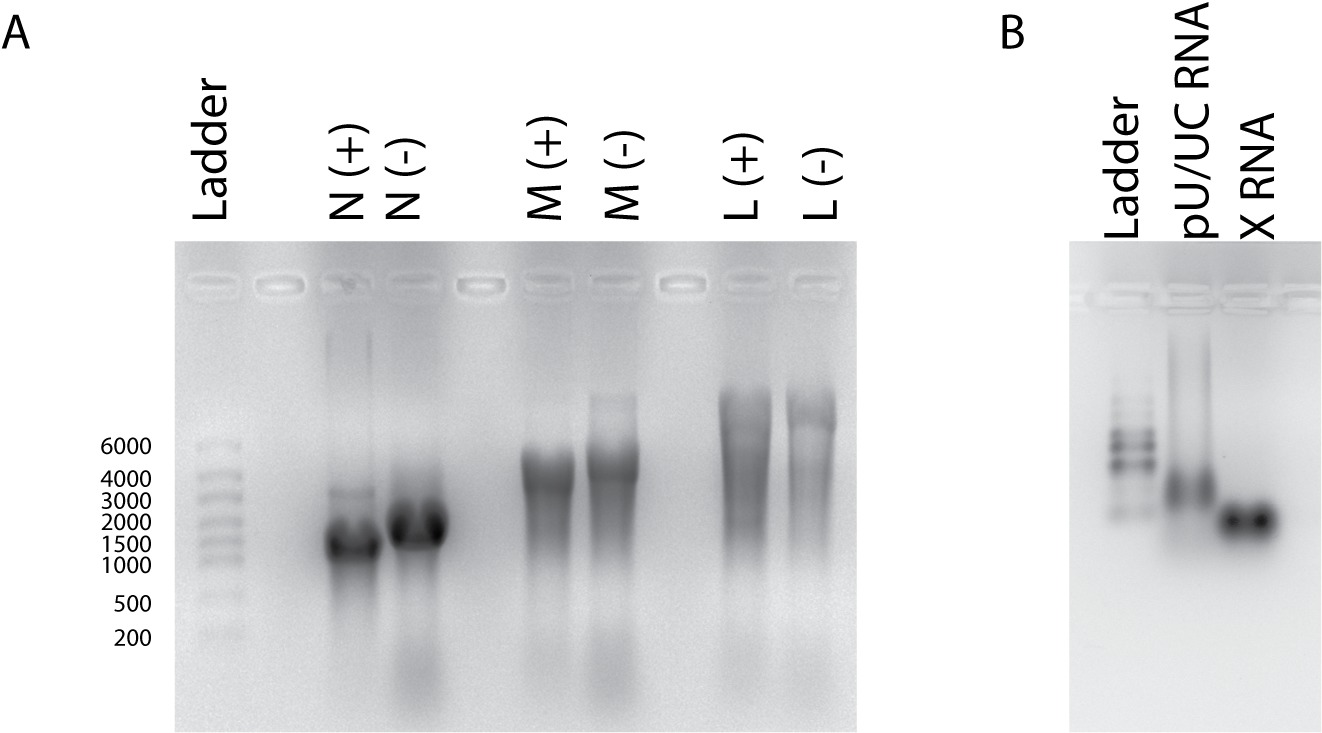

Supplement: S1 Fig — (A) IVT RNA from HTNV segment DNA templates run on a denaturing agarose gel. (+) denotes positive sense transcript and (-) denotes negative sense transcript. (B) IVT pU/UC and X RNA sequences from hepatitis C virus prepared from DNA oligonucleotides and run on a denaturing agarose gel. (TIF) [file ppat.1008483.s001.tif]

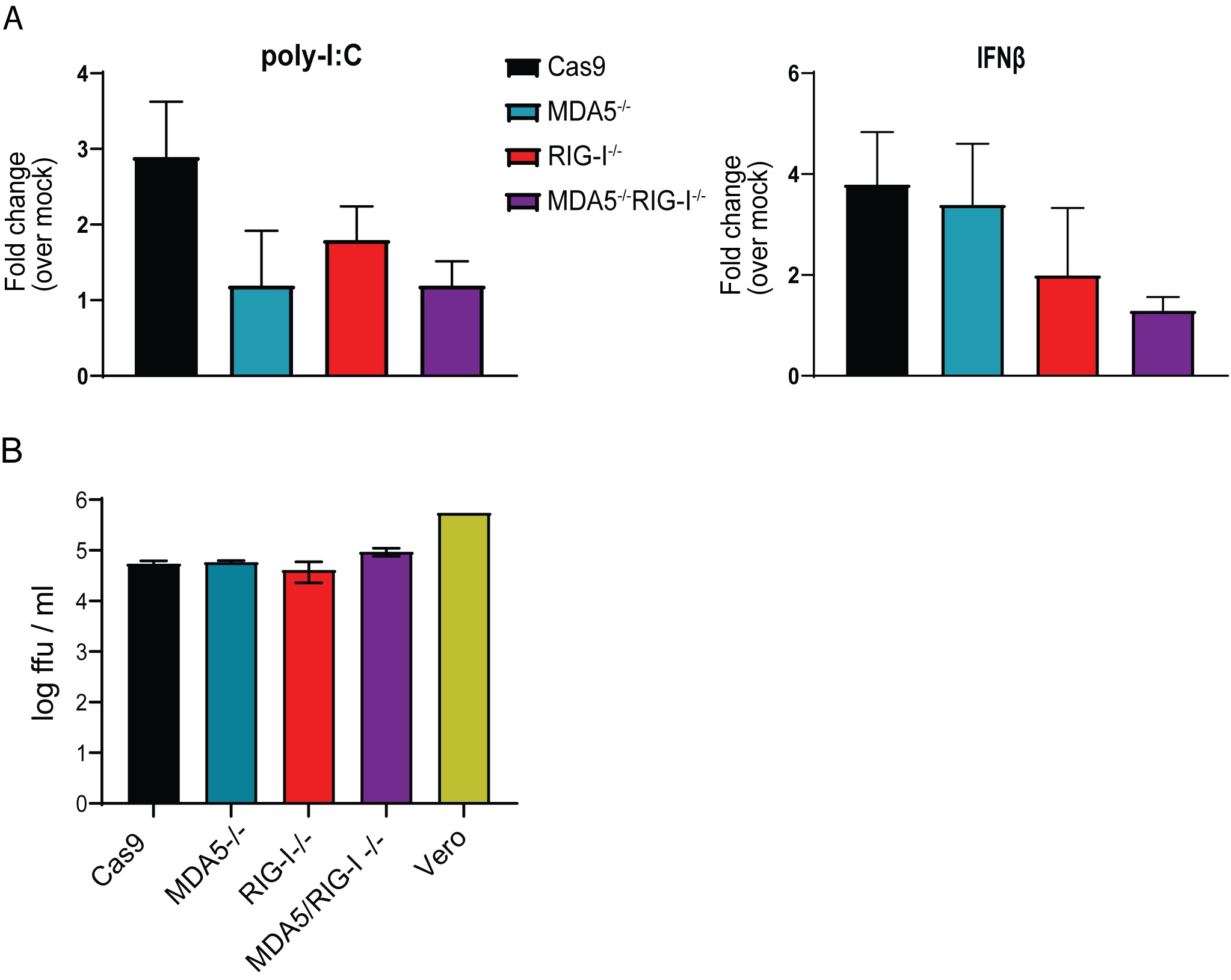

Supplement: S2 Fig — (A) RT-PCR for Il29 in HUVEC CRISPR lines treated with 1 pmol pI:C or 10U/mL IFNβ for 18hrs (±SD). (B) Titer for HTNV virus stock on HUV-EC-C CRISPR lines and Vero E6 cells. Data represent three independent experiments. Statistical analysis performed by two-way ANOVA with Prism 8 software. (TIF) [file ppat.1008483.s002.tif]

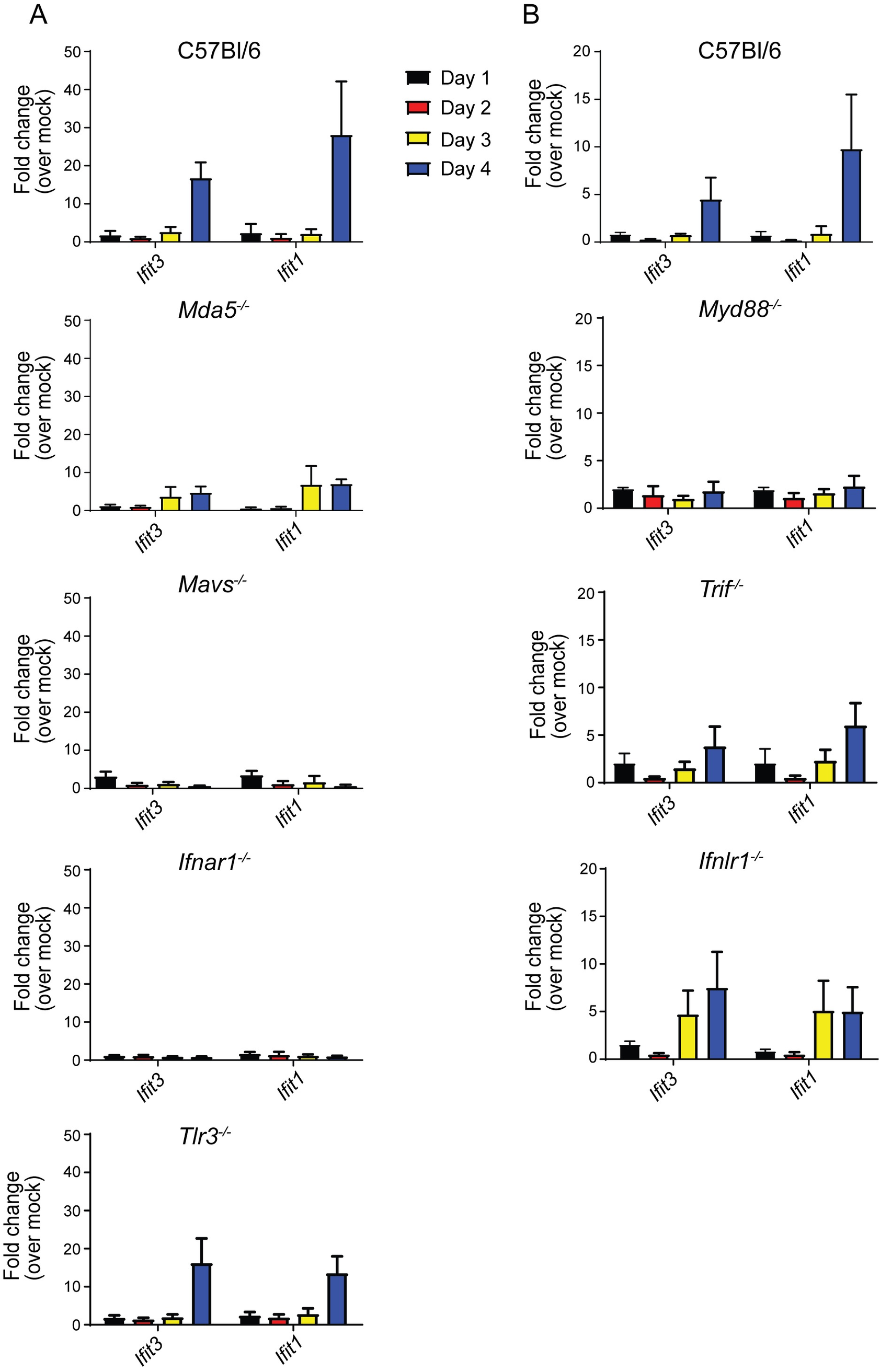

Supplement: S3 Fig — RT-PCR for Ifit3 and Ifit1 antiviral genes in WT and transgenic MEFs analyzed daily for four days post-infection with HTNV MOI 1 (±SD). (A) and (B) represent pooled data from different sets of triplicate experiments. (TIF) [file ppat.1008483.s003.tif]
